# Metronomic oral cyclosphosphamide as third-line systemic treatment or beyond in patients with inoperable locoregionally advanced recurrent or metastatic nasopharyngeal carcinoma

**DOI:** 10.1097/MD.0000000000006518

**Published:** 2017-04-14

**Authors:** Victor H.F. Lee, Dora L.W. Kwong, Ka-On Lam, Yu-Ching Lai, Yun Li, Chi-Chung Tong, Patty P.Y. Ho, Wing-Lok Chan, Lai-San Wong, Dennis K.C. Leung, Sum-Yin Chan, Fong-Ting Chan, To-Wai Leung, Anne W.M. Lee

**Affiliations:** Department of Clinical Oncology, Li Ka Shing Faculty of Medicine, The University of Hong Kong, Hong Kong.

**Keywords:** metastatic, metronomic, nasopharyngeal carcinoma, oral cyclophosphamide, recurrent

## Abstract

There is no standard third-line or further systemic treatment for patients with inoperable locoregionally advanced recurrent or metastatic nasopharyngeal carcinoma (NPC). Metronomic oral cyclophosphamide provides an acceptable and cheap option for these heavily pretreated patients who had limited choices. We conducted a prospective phase II single-arm open-label study of metronomic oral cyclophosphamide. Patients with locoregionally advanced recurrent inoperable (rT3/T4, rN2-N3b) or metastatic (rM1) NPC who had Eastern Cooperative Oncology Group (ECOG) performance status (PS) (0–2) and had progressed after at least 2 lines of palliative systemic chemotherapy were eligible. They received oral cyclophosphamide between 50 and 150 mg once daily until progressive disease or unacceptable toxicity. Objective response rate (ORR), disease control rate (DCR), biochemical response (two consecutive declines of plasma EBV DNA after treatment), progression-free survival (PFS), overall survival (OS), and safety profiles were evaluated. A total of 56 patients were recruited. Thirty-three, 13, 6, 3, and 1 patients received cyclophosphamide as 3rd, 4th, 5th, 6th, and 7th line of therapy respectively. After a median follow-up of 9.95 months (range 1.76–59.51 months), the ORR was 8.9% and the DCR was 57.1%. The median PFS and OS were 4.47 and 9.20 months, respectively. Those with PS 1 had longer median PFS (5.49 months) compared to those with PS 2 (3.75 months, *P* = .011). Besides, those who had locoregionally recurrent disease had better PFS (8.97 months, 95% CI, 0.53–17.41 months) compared to those who had distant metastases (4.14 months, 95% CI, 2.53–5.75 months, *P* = .020). Multivariable analysis revealed that PS 1 (vs 2) (*P* = .020) and locoregional recurrence (vs metastasis) (*P* = .029) were the only significant independent prognostic factors of PFS. Around 16 (28.6%) patients developed grade ≥3 adverse events, including malaise (5.4%), hematological (8.9%), gastrointestinal (3.6%), feverish (3.6%), and hemorrhagic (1.8%) events. The median cost of the whole drug treatment was 51.65 US dollars (USD) (range 4.15–142.75 USD) (1 USD = 7.8 HK dollars [HKD]). Metronomic oral cyclophosphamide is an acceptable third-line or beyond systemic therapy for locoregionally advanced recurrent or metastatic NPC with acceptable toxicity and limited financial burden.

## Abstract 2

Supplemental Digital Content is available in the text

## 1 Introduction

Undifferentiated nasopharyngeal carcinoma (NPC) is an endemic malignancy with a high incidence in Southern China, Hong Kong, Taiwan, and Singapore.^[1]^ Radiotherapy is the mainstay of treatment for early stage NPC, while concurrent chemoradiation with or without adjunct chemotherapy is indicated for locoregionally advanced disease.^[2]^ Nevertheless, about 30% of cases relapse locoregionally or distantly, despite intensive definitive treatment.^[3]^ Salvage surgery or second-course radical radiotherapy with or without chemotherapy can achieve durable disease control and promising survival for locoregional relapse.^[4,5]^ However for those with locoregionally advanced recurrent disease who had received 2 courses of radical radiotherapy or those with distant metastases, systemic chemotherapy would be the only treatment of choice. Platinum-based doublet chemotherapy including cisplatin with 5-fluorouracil, capecitabine, gemcitabine, or taxane is regarded as the standard first-line treatment due to its long-standing history and experience, especially for chemo-naïve patients.^[6]^ For second-line treatment of metastasis, whether platinum-based chemotherapy was given previously is a consideration. For patients treated with platinum-based chemotherapy, subsequent treatment depends on performance status, toxicity, and the time interval to recurrence after previous platinum-based regimen. Re-challenge with cisplatin and 5-fluorouracil can be considered in patients who enjoyed a good initial response to the same regimen with an intervening disease-free period of more than 1 year. Carboplatin may be an acceptable alternative, producing similar responses and outcomes when cisplatin is contraindicated, though it generally brings more hematological toxicities. For patients who fail platinum and 5-fluorouracil or whose diseases relapse within a year of such regimen, second-line treatment including gemcitabine, capecitabine, or taxanes with or without platinum can be considered. However, so far there has been no recognized standard third-line systemic treatment. Metronomic oral chemotherapy may provide an ideal choice to patients treated in this setting by shifting the targets from tumor cells to tumor vasculature so as to reduce the chance of drug resistance as well as offering a relatively low toxicity profile to them who have been significantly jeopardized by the long-term complications brought by prior courses of radiation therapy, surgery and chemotherapy.^[7–9]^ We presented the results of a phase II single-institution trial on the use of metronomic open-label oral cyclophosphamide as third-line treatment or beyond in patients with inoperable locoregionally advanced recurrent or metastatic NPC who had failed at least 2 lines of prior systemic chemotherapy.

## 2 Methods and materials

### 2.1 Patients

The study was approved by local institutional review board (Institutional Review Board of the University of Hong Kong/Hospital Authority West Cluster) before commencement. It was also registered with clinicaltrials.gov (NCT02794077) and conducted according to Declaration of Helsinki with good clinical practice. The study recruitment period started from January 2008 till November 2015. Patients with inoperable locoregionally advanced recurrent NPC of undifferentiated type beyond curative surgical resection or second and subsequent courses of radical radiotherapy or metastatic disease who all had received at least 2 lines of palliative systemic chemotherapy (of which one of them must be platinum-based chemotherapy) were eligible to participate into this study. All of them must have adequate hematological (absolute neutrophil count ≥1.5 × 10^9^/L; hemoglobin ≥9.0 g/dL and platelet ≥100 × 10^9^/L), renal (serum creatinine ≤1.5 × upper normal limit [ULN]) and hepatic reserves (serum bilirubin ≤1.5 × ULN; alanine aminotransferase (ALT) and aspartate aminotransferase (AST) ≤3 × ULN for patients without liver metastases or ≤5 × ULN for those with liver metastases). Patients whose Eastern Cooperative Oncology Group (ECOG) performance status (PS) was 3 or above were excluded. After written informed consent, they had baseline investigations including serum hematology, serum renal and liver biochemistry, plasma Epstein–Barr virus (EBV) deoxyribonucleic acid (DNA), as well as contrast-enhanced computed tomography (CT) scan of the head and neck, thorax and abdomen. Then they received open-label oral cyclophosphamide at 50 to 150 mg daily continuously until radiologically documented progressive disease, unacceptable toxicities or patient withdrawal. The starting dose of cyclophosphamide was determined by the treating oncologists based on patients’ performance status and their disease status. Serum hematology and biochemistry were monitored at least once every 3 weeks to monitor any treatment-related hematological and biochemical toxicities. As cyclophosphamide was in the form of 50 mg tablet, dose escalation or reduction would be a 50-mg-increment or decrement, respectively. For those starting at 50 mg daily or 100 mg daily, dose escalation to one or two dose levels up until 150 mg daily was allowed if they do not experience any treatment-related grade ≥2 events (based on Common Terminology Criteria for Adverse Events [CTCAE] version 3.0) after starting cyclophosphamide for 2 weeks. For those who developed grade ≥3 treatment-related adverse events, cyclophosphamide would be withheld until it returned to grade ≤1. They would then receive cyclophosphamide one dose level down upon resuming it. The lowest acceptable daily dose was 50 mg daily. They would also be permanently discontinued from cyclophosphamide if the adverse event(s) did not return to grade ≤1 within 21 days of treatment interruption. For those who interrupted cyclophosphamide for more than 21 days for whatever reasons, they would be permanently discontinued from the study as well. Serial blood test for plasma EBV DNA was monitored every 9 weeks while interval contrast-enhanced computed tomography (CT) scan of the head and neck, thorax and abdomen followed by interval scans were performed every 3 months for every recruited patient after commencement of study medication until progressive disease. Best objective response was determined by Response Evaluation Criteria for Solid Tumors (RECIST) 1.1.^[10]^

### 2.2 Statistical analysis

The primary study endpoint was progression-free survival (PFS), calculated from the date of start of cyclophosphamide to the date of radiologically documented progressive disease or the date of death. Secondary endpoints include objective response rate (ORR), disease control rate (DCR), biochemical PFS (calculated from the date of start of cyclophosphamide to the date of the second consecutive elevation of plasma EBV DNA from nadir after starting cyclophosphamide), overall survival (OS) (calculated from the date of start of cyclophosphamide to the date of death from any cause) and toxicity profile. Nonparametric variables were compared by Mann Whitney-*U* tests. Kaplan–Meier methods were employed for calculation of PFS and OS. Log-rank tests were employed for subgroup survival comparisons. Cox proportional hazard models with univariable and multivariable analyses were performed for prognostic factors of PFS and OS. Statistical significance was defined as *P* < .05 (two-sided). All statistical analyses were performed by Statistical Package for Social Sciences version 22. Data cut-off was performed on 1st May 2016.

## 3 Results

### 3.1 Patient characteristics

A total of 56 patients were recruited into this study with the baseline demographics shown in Table 1. Eleven (19.6%) patients had incurable locoregionally recurrent disease while 45 (80.4%) patients had distant metastases before cyclophosphamide commencement. Sixteen and 37 (66.1%) had ECOG PS 1 and 2, respectively. Thirty-three, 13, 6, 3, and 1 patients received cyclophosphamide as third, fourth, fifth, sixth, and seventh line of therapy, respectively. One (1.8%), 17 (30.4%), and 38 (67.9%) patients received 50 mg, 100 mg, and 150 mg daily as the starting dose.

**Table 1:**
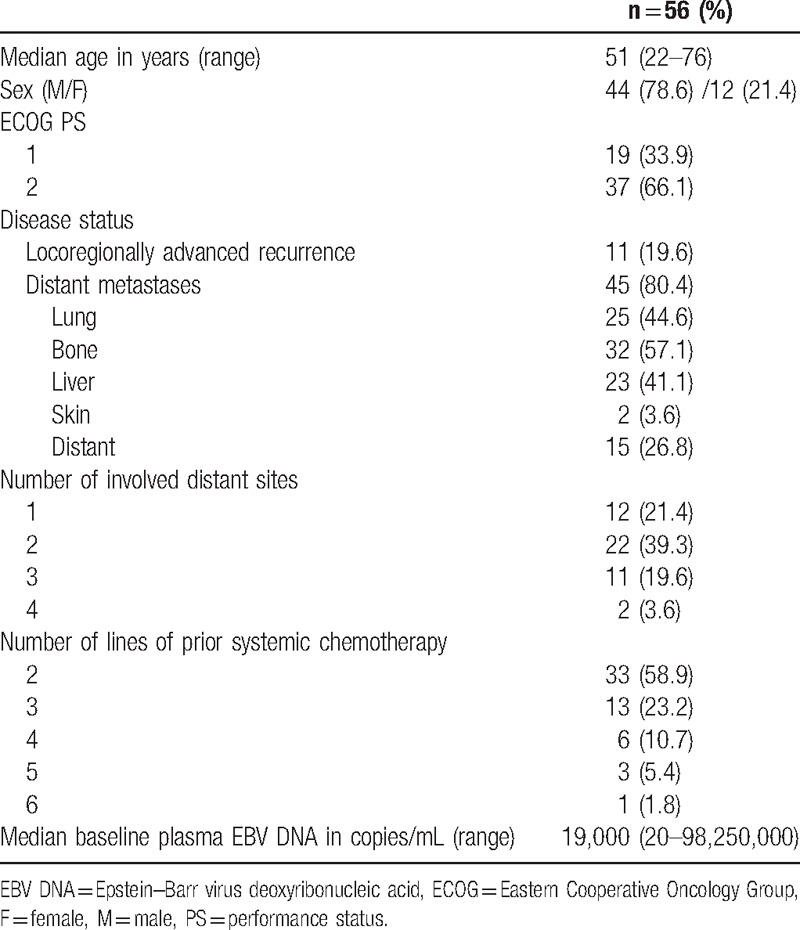
Patient characteristics.

### 3.2 Treatment efficacy and cost

**Figure 1:**
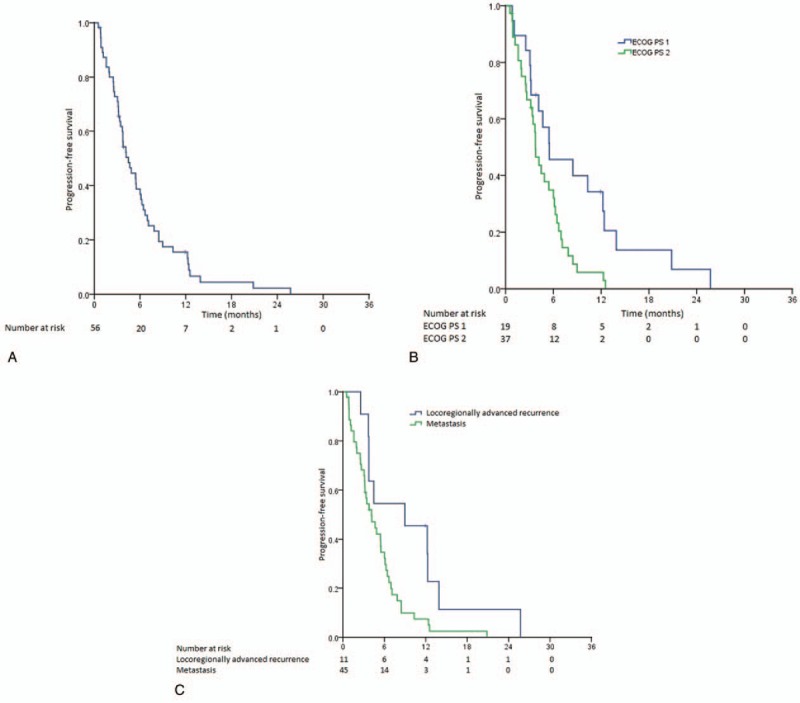
(A) Kaplan–Meier curve showing the progression-free survival of the whole study population. (B) Kaplan–Meier curves showing the progression-free survival of the study patients stratified by performance status. (B) Kaplan–Meier curves showing the progression-free survival of the study patients stratified by disease status of recurrence versus distant metastasis.

**Figure 2:**
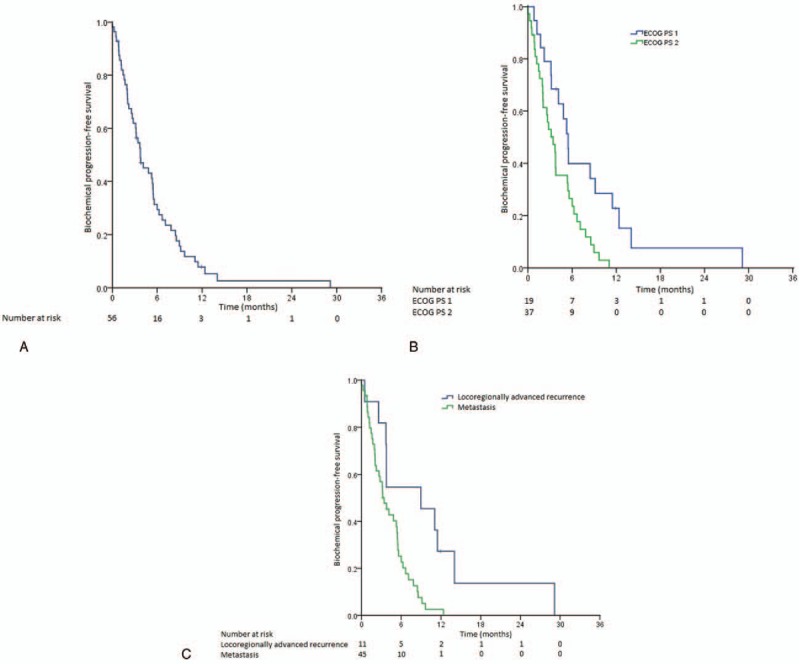
(A) Kaplan–Meir curve showing the biochemical progression-free survival of the whole study population. (B) Kaplan–Meier curves showing the biochemical progression-free survival of the study patients stratified by performance status. (C) Kaplan–Meier curves showing the biochemical progression-free survival of the study patients stratified by disease status of recurrence versus distant metastasis.

After a median follow-up duration of 9.95 months (range 1.76–59.51 months), 5 patients derived an objective response, giving an ORR of 8.9%. The DCR was 57.1%, observed in 32 patients. The median duration of therapy was 2.86 months (range 0.46–15.84 months). The median PFS for the whole study population was 4.47 months (95% confidence interval [CI], 3.18–5.76 months) (Fig. 1A). Those with ECOG PS 1 had a longer median PFS (5.49 months, 95% CI, 0.42–10.56 months) compared to those with ECOG PS 2 (3.75 months, 95% CI, 3.05–4.45 months, *P* = .011) (Fig. 1B). In addition, those who had locoregionally advanced recurrent disease had better median PFS (8.97 months, 95% CI, 0.53–17.41 months) compared to those who suffered from distant metastases (4.14 months, 95% CI, 2.53–5.75 months, *P* = .020) (Fig. 1C). The biochemical PFS for the whole study population was 3.75 months (95% CI 2.11–5.38 months) (Fig. 2A). Those who had better PS 1 enjoyed a longer median biochemical PFS (5.45 months, 95% CI, 4.57–6.34 months) than patients whose ECOG PS was 2 (3.42 months, 95% CI, 1.97–4.87, *P* = .007) (Fig. 2B). Similarly, those who had locoregionally advanced recurrent disease enjoyed longer biochemical PFS (8.97 months, 95% CI, 3.01–14.92 months) as compared to those who had distant metastases (3.42 months, 95% CI, 2.01–4.83 months; *P* = .004) (Fig. 2C).

One patient is still receiving cyclosphosphamide with stable disease at the time of submission of this publication. The cost of each 50 mg tablet cyclophosphamide was 0.30 US dollars (USD) (as on May 31st, 2016, assuming 1 USD = 7.8 Hong Kong dollars [HKD]) and thus the median cost of the whole drug treatment was 25.77 USD (range 4.15–142.75).

### 3.3 Univariable and multivariable analyses for prognostic factors of PFS and biochemical PFS

Univariable and multivariable analysis for the prognostic factors of PFS and biochemical PFS were displayed in Table 2. Multivariable analysis revealed that ECOG PS 1 (vs 2) (*P* = .020) and locoregionally advanced recurrence (vs metastasis) (*P* = .029) were the only significant independent prognostic factors of PFS. They were also the only significant independent prognostic factors for biochemical PFS (*P* = .014 and *P* = .005, respectively).

**Table 2:**
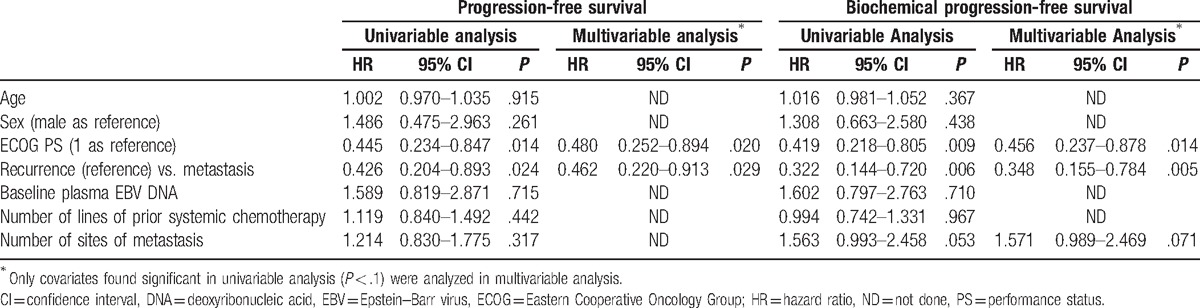
Univariable and multivariable analyses for prognostic factors of progression-free survival and biochemical progression-free survival.

### 3.4 Safety profiles

Three (5.4%) patients had dose escalation from 100 to 150 mg daily in view of good drug tolerability and absence of acute grade 2 adverse events after 2 weeks of drug therapy. However, 1 of them had subsequent dose reduction to 100 mg daily because persistent grade 3 malaise.

Adverse events were observed in 34 (60.7%) patients (Table 3). Sixteen (28.6%) patients developed grade ≥3 adverse events, including malaise (5.4%), hematological (8.9%), gastrointestinal (3.6%) and feverish (3.6%) and hemorrhagic (1.8%) events. Treatment interruption secondary to adverse events were observed in 25 (44.6%) patients. Dose reduction was necessary in 23 (41.1%) patients because of these grade ≥3 adverse events. All but 3 patients had only one level dose reduction due to their adverse events. Another 2 (3.6%) patients were permanently discontinued from cyclophosphamide because of persistent unresolving grade 3 malaise for more than 3 weeks, though it subsided completely without sequelae after cyclophosphamide termination. One patient died of sudden massive epistaxis due to bleeding recurrent tumor despite an initial response. There was no treatment-related fatality.

**Table 3:**
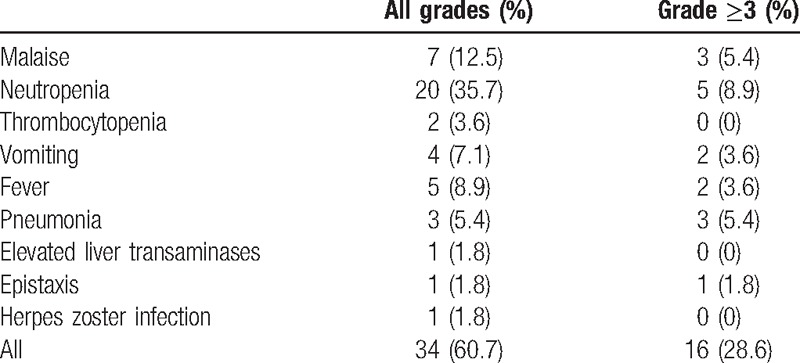
Toxicity profiles.

### 3.5 Postcyclophosphamide systemic treatment

**Figure 3:**
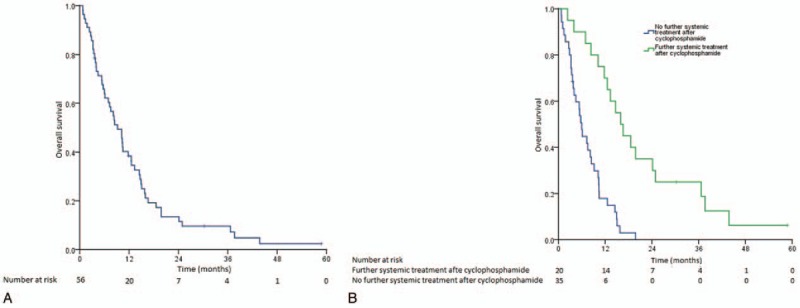
(A) Kaplan–Meier curve showing the overall survival of the whole study population. (B) Kaplan–Meier curves showing the overall survival of the study patients stratified by the use of further systemic treatment after cyclophosphamide.

Twenty (35.7%) patients received further systemic treatment after progression to cyclophosphamide. The lines of further systemic treatment ranged from 1 to 9 (median 2). The median OS for the whole study population was 9.20 months (95% CI, 6.32–12.08 months) (Fig. 3A). Those with locoregionally advanced recurrence (14.49 months, 95% CI, 10.56–18.42 months) tended to survive longer than those with distant metastasis (8.35 months, 95% CI, 5.89–10.80; *P* = .099). Those who received further systemic treatment enjoyed a longer median OS (15.97 months, 95% CI 11.72–20.22 months) than those who did not (5.98 months, 95% CI, 4.92–7.04 months, *P* < .001) (Fig. 3B). The number of lines of postcyclophosphamide systemic treatment was prognostic of OS in univariable (*P* = .001) and multivariable analysis (*P* < .001). (Supplementary Table 1).

## 4 Discussion

The survival outcomes of patients with previously untreated NPC have improved steadily for the past few decades, owing to significant contributions by use of concurrent chemoradiation with or without adjunct chemotherapy and the implementation of precision radiation techniques including intensity-modulated radiation therapy. Nevertheless, still between 5% and 15% of these patients will eventually develop locoregional failure and 15% to 30% will fail distantly. A vast majority of these patients with locoregional recurrence still have to resort to palliative systemic chemotherapy after failure to salvage surgery or re-irradiation. Systemic chemotherapy remains the mainstay of treatment for these patients with inoperable locoregionally advanced recurrent and those metastatic NPC. For chemo-naïve patients, monotherapy may be only recommended for those patients with suboptimal PS as the response rate and survival outcomes are less than satisfactory (Supplementary Table 2).^[11–23]^ Platinum doublet chemotherapy is still recommended as the first-line treatment for those who have satisfactory PS, owing to the platinum sensitivity and long-standing history in clinical use (Supplementary Table 3).^[12,24–52]^ In particular, cisplatin and 5-fluorouracil is the most popular selection due to its widespread use in head and neck squamous cell carcinoma and acceptable toxicity. Recently, newer agents including paclitaxel, docetaxel, gemcitabine and capecitabine have gradually replaced 5-fluorouracil as the companion of cisplatin, so as to avoid prolonged hospitalization for 5-fluorouracil infusion. Meanwhile newer platinum compounds including carboplatin, oxaliplatin, nedaplatin, and lobaplatin (both manufactured in China) were tested as alternative to cisplatin for their more favorable toxicity profile of nephrotoxicity and neurotoxicity. Nevertheless, cisplatin is still preferred to other platinum compounds as 2 old randomized-controlled trials on head and neck cancers showed a superior response rate and survival outcomes with cisplatin. Polychemotherapy, though theoretically more potent, is also more toxic and distressing to patients and thus not routinely recommended (Supplementary Table 4).^[53–64]^ Choice of second-line systemic chemotherapy heavily depends on the drugs used in the first line. For patients treated with prior platinum-based chemotherapy, subsequent treatment depends on performance status, toxicity, and the interval to recurrence after the last platinum-based regimen. Re-challenge with cisplatin and 5-fluorouracil can be considered in patients who enjoyed a good initial response to the same regimen with a relapse-free period of more than 1 year. Carboplatin is an acceptable substitute producing similar responses and outcomes when cisplatin is contraindicated, though it generally gives rise to more hematological toxicities. For patients who fail platinum and 5-fluorouracil or whose disease relapse within a year of such a regimen, second-line treatment including gemcitabine, capecitabine, or taxanes with or without platinum is generally recommended. There is no standard third-line treatment as virtually no publication has addressed this issue. Patients after failure to 2 prior lines of treatment are generally physically compromised, as brought by the permanent platinum-related side effects including nephrotoxicity, neurotoxicity and immunosuppression. However, the relatively slow disease tempo of NPC as compared to other common solid malignancies can be distressing and torturing to patients for quite a while secondary to the intracranial symptoms of headache, facial paresthesia, diplopia by the locally advanced recurrence and the emerging side effects after salvage surgery and 2nd course radiotherapy including trismus, temporal lobe necrosis, osteoradionecrosis, poor oral hygiene, dysphagia, etc.

Oral metronomic chemotherapy may provide promising disease control and symptom relief for these heavily pretreated patients, while maintaining a relatively reasonable quality of life with less devastating toxicities compared to the intravenous drugs. Metronomic chemotherapy was first described by Hanahan et al,^[65]^ which refers to the close and regular administration of chemotherapy for a long period of time without an intended drug-free interval. This idea was developed to overcome the drug resistance by shifting the therapeutic target from the tumor cells to the tumor vasculature.^[9,66]^ Standard chemotherapy cycles and schedules only cause meager endothelial cell damage. These cells can easily repair during the rest periods of chemotherapy and thus continue to support growth of tumor cells leading to eventually drug resistance. The more compact administration of low-dose chemotherapy may sound more effective against tumor vasculature while giving less toxicities to patients and avoiding unnecessary drug interruption. In fact, Browder and Kerbel first highlighted the anti-angiogenic phenomenon after metronomic scheduling of cyclophosphamide, more effective than the conventional scheduling of chemotherapy in overcoming drug resistance in breast cancer cell lines.^[67,68]^ Other plausible mechanisms of metronomic chemotherapy include activation of immunity through reduction of regulatory T-cells and dendritic cell maturation and direct tumor cell kill. Previous studies have clearly demonstrated the efficacy and safety of oral metronomic cyclophosphamide, used either as monotherapy, or in combination with other chemotherapeutic agents or targeted drugs in various types of solid malignancies including breast cancer, prostate cancer, colorectal cancer, ovarian cancer and melanoma, giving an objective response rate between 5% and 60% and time to progression between 1.8 and 7.2 months.^[69–73]^ Perhaps and more important to patients, cyclophosphamide even without financial reimbursement is not costly to patients who take it for a prolonged time period.

Recently targeted therapy including antiepidermal growth factor receptor (EGFR), antivascular endothelial growth factor (VEGF) and multikinase inhibitor becomes an alternative option for those who are refractory or refuses chemotherapy.^[74–83]^ Most of them, however, only provide a modest and short response. In particular, sorafenib and sunitinib can give rise to serious and fatal hemorrhage events.^[81]^ In addition, immunotherapy has also evolved gradually in the treatment of recurrent/metastatic NPC. The immunological approach encompasses various strategies namely EBV-directed adoptive and active immunotherapy, administration of antibodies, induction of EBV lytic cycle, and immune checkpoint inhibition.^[84–92]^ Though preliminary results are encouraging and safe, these approaches are still experimental and only limited to tertiary institutions with expertise and comprehensive laboratory infrastructure. Immune checkpoint inhibitors against programmed cell death-1 (PD-1) have been recently extensively investigated for recurrent/metastatic NPC. A phase Ib study demonstrated that pembrolizumab gave an OR rate of 22.2% and a disease control rate of 77.8% in 27 heavily pretreated patients with advanced NPC.^[93]^ Phase II trials have been ongoing to further investigate the efficacy and safety of pembrolizumab (NCT02611960) and nivolumab (NCT02339558) as second or subsequent line treatment.

In summary, metronomic oral cyclosphosphamide is an acceptable 3rd line systemic treatment for inoperable recurrent or metastatic NPC, which provides encouraging disease control, reasonable toxicity and affordable financial burden.

## Supplementary Material

Supplemental Digital Content
