# Supplementary material for: Metronomic oral cyclosphosphamide as third-line systemic treatment or beyond in patients with inoperable locoregionally advanced recurrent or metastatic nasopharyngeal carcinoma
Source: Medicine (Baltimore). 2017 Apr 14;96(15):e6518. doi: 10.1097/MD.0000000000006518 (PMC5403079; doi:10.1097/MD.0000000000006518)
Supplement: Supplemental Digital Content [file medi-96-e6518-s001.doc]

**Supplementary TABLE 1.** Univariable and Multivariable Analysis For Prognostic Factors of Overall Survival.

|  | **Overall survival** | | | | | |
| --- | --- | --- | --- | --- | --- | --- |
|  | **Univariable Analysis** | | | **Multivariable Analysis*** | | |
|  | HR | 95% CI | *P* | HR | 95% CI | *P* |
| Age | 1.001 | 0.966–1.036 | 0.973 | ND | | |
| Sex (male as reference) | 1.309 | 0.668–2.567 | 0.433 | ND | | |
| ECOG PS (1 as reference) | 0.700 | 0.391–1.256 | 0.232 | ND | | |
| Recurrence (reference) vs. metastasis | 0.549 | 0.265–1.134 | 0.105 | ND | | |
| Baseline plasma EBV DNA | 1.632 | 0.779–2.568 | 0.476 | ND | | |
| Number of lines of prior systemic chemotherapy | 1.229 | 0.909–1.661 | 0.181 | ND | | |
| Number of sites of distant metastasis | 1.252 | 0.870–1.802 | 0.226 | ND | | |
| Number of lines of post-cyclophosphamide systemic treatment | 1.805 | 1.285–2.538 | 0.001 | 1.856 | 1.304–2.645 | <0.001 |

*Only covariates found significant in univariable analysis (*P* < 0.1) were analyzed in multivariable analysis.

CI = confidence interval, DNA = deoxyribonucleic acid, EBV = Epstein-Barr virus, ECOG = Eastern Cooperative Oncology Group, HR = hazard ratio, ND = not done, PS = performance status.

**Supplementary TABLE 2.** Monochemotherapy in Recurrent and/or Metastatic Nasopharyngeal Carcinoma as First-line and/or Subsequent Line Setting.

| Study | Setting | Phase | Number of Patients | Regimen | OR Rate (%) | CR Rate (%) | Median PFS | Median OS |
| --- | --- | --- | --- | --- | --- | --- | --- | --- |
| 1^st^ line or beyond | | | | | | | | |
| Foo^11^ | 1^st^ line or beyond | Phase II | 25 M pretreated  27 M untreated | G | 28  48 | 4  3.7 | 3.6 months  5.1 months | 7.2 months  10.5 months |
| Ma^12^ | 1^st^ line or beyond | Retrospective | 18 R + M | G | 34 | 6 | 31% (1-month) | 48% (1-year) |
| 2^nd^ line or beyond | | | | | | | | |
| Dugan^13^ | 2^nd^ line or beyond | Phase II | 108 R + M | MIT | 25 | NR | 4.5 months | 13 months |
| Au^14^ | 2^nd^ line or beyond | Phase II | 24 M | PAC | 21.7 | 0 | 7.5 months | 12 months |
| Poon^15^ | 2^nd^ line or beyond | Phase II | 28 M | IRI | 14 | 0 | 3.9 months | 11.4 months |
| Chua^16^ | 2^nd^ line | Phase II | 17 R + M | CAP | 23.5 | 5.9 | 4.9 months | 7.6 months |
| Chua^17^ | 2^nd^ line or beyond | Retrospective | 49 R + M | CAP | 37 | 6 | 5 months | 14 months; 54% (1-year) |
| Ciuleanu^18^ | 2^nd^ line or beyond | Phase II | 26 R + M | CAP | 48 | 9 | 14 months | 62% (1-year) |
| Zhang^19^ | 2^nd^ line or beyond | Phase II | 32 R + M | G | 43.8 | 0 | 5.1 months | 16 months; 63% (1-year) |
| Ngeow^20^ | 2^nd^ line or beyond | Phase II | 30 R + M | DOC (weekly) | 37 | 0 | 5.3 months | 12.8 months |
| Zhang^21^ | 2^nd^ line or beyond | Phase II | 35 R + M | PEM | 2.9 | 0 | 1.5 months | 13.3 months |
| Peng^22^ | 2^nd^ line or beyond | Retrospective | 39 R + M | S-1 | 30.7 | 2.6 | 5.6 months (median TTP) | 13.9 months |
| Tsao^23^ | 2^nd^ line | Phase II | 13 M | TAS-106 | 0 | 0 | 48 days | 280 days |
| Current | 3^rd^ line or beyond | Phase II | 11 R  45 M | CYC | 8.9 | 0 | 9.0 months  4.1 months | 14.5 months  8.4 months |

CAP = capecitabine, CR = complete response, CYC = cyclophosphamide, DOC = docetaxel, G = gemcitabine, IRI = irinotecan, M = metastatic, MIT = mitoxantrone, NR = not reported, OR = objective response, OS = overall survival, PAC = paclitaxel, PEM = pemetrexed, PFS = progression-free survival, R = recurrent, TTP = time to progression.

**Supplementary TABLE 3.** Doublet Chemotherapy Regimens in Recurrent and/or Metastatic Nasopharyngeal Carcinoma as First-line or Subsequent Line Setting.

| Study | Setting | Phase | Number of Patients | Regimen | OR Rate (%) | CR Rate (%) | Median PFS | Median OS |
| --- | --- | --- | --- | --- | --- | --- | --- | --- |
| 1^st^ line or beyond | | | | | | | | |
| Wang^24^ | UNK | Retrospective | 25 M | P + F | 76 | 8 | NR | NR |
| Au^25^ | 1^st^ line | Phase II | 24 R + M | P + F | 66 | 13 | 8 months | 11 months |
| Stein^26^ | 1^st^ line | Phase II | 18 R + M | P + I | 59 | 15 | NR | NR |
| Yeo^27^ | 1^st^ line or beyond | Phase II | 42 M | C + F | 38 | 17 | NR | 12.1 months |
| Yeo^28^ | 1^st^ line or beyond | Phase II | 27 R + M | C + PAC | 59 | 11 | 6 months | 13.9 months |
| Tan^29^ | 1^st^ line | Phase II | 32 M | C + PAC | 75 | 3 | 7 months | 12 months |
| Ciuleanu^30^ | 1^st^ line | Phase II | 40 M | C + PAC | 27.5 | 7.5 | 3.5 months | 11.5 months |
| Ngan^31^ | 1st line or beyond | Phase II | 44 R + M | P + G | 73 | 20 | 10.6 months | 15 months |
| Wang^32^ | 1^st^ line or beyond | Retrospective | 75 R + M | P + G | 42.7 | 5.3 | 5.6 months | 9 months |
| Ma^12^ | 1^st^ line or beyond | Phase II | 14 R + M | P + G | 64 | 14 | 13% (1-year) | 68% (1-year) |
| Ma^33^ | 1^st^ line | Phase II | 40 R + M | O + G | 56.1 | 0 | 9 months | 19.6 months |
| McCarthy^34^ | 1^st^ line | Phase II | 9 R + M | P + DOC | 22 | 0 | 8.4 months | 76% (1-year) |
| Chua^35^ | 1^st^ line | Phase II | 19 M | P + DOC | 62.5 | 6.3 | 5.6 months | 12.4 months |
| Li^36^ | 1^st^ line | Phase II | 48 M | P + CAP | 62.5 | 6.3 | 7.7 months | 13.3 months |
| Huang^37^ | 1^st^ line or beyond | Phase II | 34 R + M | I + DOC | 67.6 | 14.7 | 6 months | NR |
| Yau^38^ | 1^st^ line or beyond | Phase II | 15 R + M | P + PEM | 20 | 7 | 30 weeks (median TTP) | NR |
| Chua^39^ | 1^st^ line | Phase II | 44 M | P + CAP | 53.8 | 2.6 | 7.3 months (median TTP) | 28.0 months |
| Jin^40^ | 1^st^ line | Retrospective | 822 R + M | P + F  PAC + P  P + G  B + P + F  PAC + P + F | 60.2  61.7  71.1  69.1  74.0 | 2.8  4.2  6.9  4.6  5.8 | 5.0 months  5.5 months  6.6 months  5.5 months  6.0 months | 19.5 months  21.0 months  21.5 months  19.0 months  21.0 months |
| Ji^41^ | 1^st^ line | Phase II | 47 R + M | DOC + P | 70.2 | 12.8 | 9.6 months | 28.5 months |
| Long^42^ | 1^st^ line or beyond | Phase II | 39 R + M | LOB + DOC | 61.5 | 7.7 | 10 months | NR |
| Zheng^43^ | 1^st^ line or beyond | Phase II | 33 M | P + NOL or  P + F | 31.3%  35.3% | 0  0 | 3.4 months (median TTP)  3.8 months  (median TTP) | 9.5 months  10.0 months |
| Peng^44^ | 1^st^ line | Phase II | 78 R + M | NED + DOC | 65.8 | 6.8 | 7.9  (median TTP) | 15.7 |
| Hsieh^45^ | 1^st^ line | Phase II | 52 R + M | P + G | 56.2 | NR | 9.8 months | 14.6 months |
| 2^nd^ line or beyond | | | | | | | | |
| Chi^46^ | 2^nd^ line or beyond | Phase II | 20 R  15 M | P + F + LV | 100  80 | 15  13 | NR  NR | 34 months  14 months |
| Chua^47^ | 2^nd^ line | Phase II | 18 R + M | I + F + LV | 56 | 6 | 6.5 months | 51% (1-year) |
| Altundag^48^ | 2^nd^ line or beyond | Phase II | 21 R + M | I + DOC | 33.3 | 0 | 7 months | NR |
| Wang^49^ | 2^nd^ line or beyond | Phase II | 39 M | G + V | 36 | 3 | 5.6 months | 11.9 months |
| Dede^50^ | 2^nd^ line or beyond | Retrospective | 30 R + M | I + D | 30 | 0 | 4 months (median TTP) | NR |
| Chen^51^ | 2^nd^ line or beyond | Phase II | 61 R + M | G + V | 37.7 | 1.6 | 5.2 months | 5.2 months |
| Peng^52^ | 2^nd^ line or beyond | Phase II | 48 R + M | NED + CAP | 41.7 | 4.2 | 5.8 months (median TTP) | 12.4 months |

B = bleomycin, CAP = capecitabine, C = carboplatin, CR = complete response, DOC = docetaxel, D = doxorubicin, F = 5-fluorouracil, G = gemcitabine, I = ifosphamide, LOB = lobaplatin, LV = leucovorin, M = metastatic, NED = nedaplatin, NOL = nolatrexed, NR = not reported, OR = objective response, OS = overall survival, O = oxaliplatin, P = cisplatin, PAC = paclitaxel, PEM = pemetrexed, PFS = progression-free survival, R = recurrent, TTP = time to progression, UNK = unknown, V = vinorelbine.

**Supplementary TABLE 4.** Results for Polychemotherapy in Recurrent and/or Metastatic Nasopharyngeal Carcinoma as First-line or Subsequent Line Setting.

| Study | Setting | Phase | Number of Patients | Regimen | OR Rate (%) | CR Rate (%) | Median PFS | Median OS |
| --- | --- | --- | --- | --- | --- | --- | --- | --- |
| Boussen^53^ | 1^st^ line or beyond | Phase II | 49 R + M | P + B + F | 79 | 19 | 50 months | NR |
| Su^54^ | 1^st^ line | Phase II | 25 R + M | P + B + F | 40 | 3 | NR | NR |
| Azli^55^ | 1^st^ line or beyond | Phase II | 44 R + M | B + E + P | 45 | 20 | 53 months | NR |
| Siu^56^ | 1st line | Phase I/II | 17 R  44 M | CAPABLE | 41  80 | 23.5  6.8 | NR  NR | 16 months  14 months |
| Taamma^57^ | 1^st^ line | Phase II | 23 R + M | F + B + E + P | 78 | 39 | 42 months | NR |
| Hasbini^58^ | 1^st^ line | Phase II | 44 R + M | F + MIC + E + P | 52 | 13 | 9 months | 14 months |
| Leong^59^ | 1^st^ line | Phase II | 28 M | C + G + PAC + F + LV | 86 | 11 | 8 months | 22 months |
| Huang^60^ | 1^st^ line or beyond | Phase II | 56 R + M | DOC + P + F | 72.5 | 9.8 | NR | NR |
| Xue^61^ | 1^st^ line | Phase II | 54 R + M | SOR + P + F | 77.8 | 1.9 | 7.2 months | 11.8 months |
| Jin^62^ | 1^st^ line | Phase II | 30 M | ENDO + P + G | 85.7 | 50 | 19.4 months | 90.2% (1-year) |
| Hsieh^63^ | 1^st^ line | Phase II | 22 R + M | P + TEG/URA + LV + MIC | 59.1 | 13.6 | 10 months (median TTP) | 16 monhts |
| 2^nd^ line or beyond | | | | | | | | |
| Chen^64^ | 2^nd^ line or beyond | Phase II | 95 R+ M | PAC + P + F | 78.9 | 0 | 9.1 months | 27.2 months |

B = bleomycin, CAPABLE = cyclophospamide + bleomycin + doxorubicin + cisplatin, C = carboplatin, CR = complete response, DOC = docetaxel, E = epirubicin, ENDO = endostar, F = 5-fluorouracil, G = gemcitabine, I = ifosphamide, LV = leucovorin, M = metastatic, MIC = mitomycin C, NR = not reported, OR = objective response, OS = overall survival, P = cisplatin, PAC = paclitaxel, PFS = progression-free survival, R = recurrent, SOR = sorafenib, TEG/URA = tegafur-uracil.
